# Impact of biological therapy on work outcomes in patients with axial spondyloarthritis: results from the British Society for Rheumatology Biologics Register (BSRBR-AS) and meta-analysis

**DOI:** 10.1136/annrheumdis-2018-213590

**Published:** 2018-08-03

**Authors:** Joanna Shim, Gareth T Jones, Ejaz M I Pathan, Gary J Macfarlane

**Affiliations:** 1 Epidemiology Group, School of Medicine, Medical Sciences and Nutrition, University of Aberdeen, Aberdeen, UK; 2 Arthritis Research UK/Medical Research Council Centre for Musculoskeletal Health and Work, Aberdeen, UK; 3 Aberdeen Centre for Arthritis and Musculoskeletal Health, University of Aberdeen, Aberdeen, UK; 4 Spondylitis Program, Department of Rheumatology, Toronto Western Hospital, University Health Network, Toronto, Ontario, Canada

**Keywords:** axial spondyloarthritis, meta-analysis, biologic therapy, work

## Abstract

**Objectives:**

To quantify, among patients with axial spondyloarthritis (axSpA), the benefit on work outcomes associated with commencing biologic therapy.

**Methods:**

The British Society for Rheumatology Biologics Register in Axial Spondyloarthritis (BSRBRAS) recruited patients meeting Assessment of SpondyloArthritis International Society criteria for axSpA naïve to biological therapy across 83 centres in Great Britain. Work outcomes (measured using the Work Productivity and Activity Impairment Index) were compared between those starting biological therapy at the time of recruitment and those not. Differences between treatment groups were adjusted using propensity score matching. Results from BSRBR-AS were combined with other studies in a meta-analysis to calculate pooled estimates.

**Results:**

Of the 577 participants in this analysis who were in employment, 27.9% were starting biological therapy at the time of recruitment. After propensity score adjustment, patients undergoing biological therapy, at 12-month follow-up, experienced significantly greater improvements (relative to non-biological therapy) in presenteeism (−9.4%, 95% CI −15.3% to –3.5%), overall work impairment (−13.9%, 95% CI −21.1% to –6.7%) and overall activity impairment (−19.2%, 95% CI −26.3% to –12.2%). There was no difference in absenteeism (−1.5%, 95% CI −8.0 to 4.9). Despite these improvements, impact on work was still greater in the biological treated cohort at follow-up. In the meta-analysis including 1109 subjects across observational studies and trials, treatment with biological therapy was associated with significantly greater improvements in presenteeism, work impairment and activity impairment, but there was no difference in absenteeism.

**Conclusions:**

There is consistent evidence that treatment with biological therapy significantly improves work productivity and activity impairment in people with axSpA. However, there remain substantial unmet needs in relation to work.

## Introduction

For working age adults, the ability to participate in working life is important from an economic standpoint and for social and psychological health.^1^ In a comprehensive review, Waddell and Burton^2^ provided a clear evidence base for the positive impact work has on health and well-being. When these benefits are disrupted by unemployment, they found subsequent poorer mental and physical health, increased morbidity and higher consultation rates.^2^ People living with axial spondyloarthritis (axSpA) have identified the ability to stay at work as a priority.^3 4^

AxSpA can lead to significant functional limitations and reduced work productivity.^4 5^ Work disability affects up to 30% of patients with ankylosing spondylitis (AS).^6–8^ Compared with the general population, patients with AS report lower employment rates and more absenteeism.^6 9 10^ Boonen and van der Linden^11^ reported that withdrawal from work was three times higher among patients with AS than observed in the general population, especially in those with physically demanding jobs.

Biological therapy leads to short-term and long-term improvements in disease activity and health-related quality of life.^12–14^ However, evidence is equivocal on whether work outcomes are improved. In a systematic review, van der Burg *et al* 15 demonstrated significant improvements in presenteeism on biological therapy, but results were inconsistent on whether they improved absenteeism and allowed patients to stay in work.^15^

This study therefore aimed to determine, using a national disease register in Great Britain whether, among patients with axSpA, biological therapy was associated with better work outcomes in comparison with those not on such therapy, and to combine the results with other studies in a meta-analysis.

## Methods

The protocol of the British Society of Rheumatology Biologics register in Axial Spondyloarthritis (BSRBR-AS) has previously been published.^16^ Briefly, patients were required to meet the Assessment of SpondyloArthritis International Society criteria for radiographic or non-radiographic axSpA at the time of recruitment and were required to be naïve to biological therapies. Eligible patients commencing on biological therapy were recruited to the ‘biological’ cohort, the remainder to the ‘non-biological’ cohort; this was based on clinical decision rather than study protocol. The biological cohort comprised patients commencing adalimumab, etanercept or certolizumab pegol. Clinical data collected by site clinicians and research nurses at routine clinical appointments were entered into electronic case report forms at recruitment (baseline) and at 12-month follow-up. Patients were invited to self-complete a questionnaire at the same time-points, which included demographic information, smoking status, alcohol consumption, disease activity, AS-related measures and quality of life.

The impact of axSpA on work outcomes was measured using the Work Productivity and Activity Impairment: Specific Health Problem questionnaire.^17^ The questionnaire has been psychometrically validated in AS^18^ and measures absenteeism (absence from paid work), presenteeism (at-work productivity loss), overall work impairment (combination of absenteeism and presenteeism, ie, reduced overall productivity) and overall activity impairment (reduced leisure activities), in relation to their disease. Higher scores, expressed as percentages, represent a worse state. Participants who were in paid employment at baseline and completed both a baseline and 12-month follow-up questionnaire were eligible for this analysis. Analysis was conducted on the June 2017 download of data. Appropriate National Health Service (NHS) Research and Development approvals were obtained for each site. All patients provided written informed consent.

### BSRBR-AS analysis

The principal outcomes of interest for this analysis were changes in self-reported absenteeism, presenteeism, overall work impairment and overall activity impairment in the biological cohort in comparison with the non-biological cohort. In clinical practice, the decision to initiate biological therapy is strongly influenced by disease activity,^19^ and therefore, the biological and non-biological cohorts represent distinct clinical populations and direct comparison would lead to confounding by indication. This was addressed by propensity matching. There is currently no strong consensus on the approach to variable selection for propensity score models,^20^ but most relevant confounders were considered in our study, and where there were issues of collinearity, variables were omitted from the model based on the variance inflation factor. Propensity scores were developed based on age, gender, smoking status and disease duration in a logistic regression model. For each individual commencing biological therapy, nearest-neighbour propensity score was used to identify an individual for matching, not starting biological therapy, by accounting for covariates that predict treatment assignment. Density distributions of the propensity scores of the two treatment groups were plotted and visually compared with ensure that the common support condition was met as per Caliendo and Kopeinig.^21^ The standardised differences and variance ratios for raw and matched observations for presenteeism, work impairment, activity impairment and absenteeism indicated that matching on the propensity score balanced the covariates: differences were close to zero and variance ratios close to one. After propensity score matching, the differences in work outcomes for the biological therapy and non-biological therapy groups were analysed. Independent-sample Student’s t-test was used for group comparisons of the changes in work outcomes. A sensitivity analyses was conducted to explore the degree to which unmeasured confounding could alter study results, using the method proposed by Rosenbaum.^22^

Data analysis was performed using STATA V.14.0.

### Systematic review and meta-analysis

The conduct and reporting of this meta-analysis was guided by the Preferred Reporting Items for Systematic Reviews and Meta-Analyses statement.^23^

Studies were eligible for inclusion based on the following criteria:

- *Population*: AxSpA by recognised criteria or clinical diagnosis.
- *Study design*: observational studies (prospective and retrospective cohort studies), randomised controlled trials (RCTs) and quasi-RCTs.
- *Outcomes*: outcomes reported included changes in work measures (eg, work status, WPAI, AS-Work Instability Scale (AS-WIS) and sick leave) for a biological treatment group versus a placebo/non-biological treatment group. Studies must have provided data in relation to this comparison or provided sufficient data to allow its calculation.

If more than one publication was identified based on the same cohort or population, a single report was selected based on the relevance of work measures reported and the largest number of subjects.

Studies were identified by searching electronic databases: Ovid MEDLINE, Embase, Evidence Based Medicine and Cochrane Library up to March 2018. Reference lists of articles were also screened for inclusion. The search strategy can be found in online supplementary appendix 1. Potentially eligible abstracts were screened by two reviewers, and any disagreements were resolved by discussion. Ten per cent of the articles excluded at full-text screening were independently reviewed by a third reviewer. One reviewer extracted relevant data from included studies, and a second reviewer cross-checked the extracted data. Disagreements were resolved by discussion among all three reviewers. Where it was considered that a study was potentially eligible but the way that the quantitative results were presented did not meet inclusion criteria, the corresponding author was contacted to determine whether the relevant data, to make the study eligible, could be provided.

10.1136/annrheumdis-2018-213590.supp1 Supplementary file 1

For continuous outcome variables, the mean difference of change in work parameters, comparing biological and non-biological therapy groups, was calculated with 95% CIs. Heterogeneity was assessed using the χ^2^ statistic and quantified by I^2^; 0% ≤ I^2^≤40% is considered to indicate minimal heterogeneity and I^2^ >40% moderate to high heterogeneity.^24^ In cases where there was no evidence of moderate to high heterogeneity, a fixed-effects model with inverse variance weighting was used to obtain an overall mean difference or effect estimate. If moderate to high heterogeneity was evident between studies, a random-effects model was adopted, and a sensitivity analysis was conducted by sequentially omitting individual studies to identify the influence of the study on the pooled outcome.

The meta-analysis was conducted using STATA V.14.0.

## Results

Within the BSRBR-AS, 972 participants completed and returned both the baseline and 12-month follow-up questionnaire, of whom 577 (59.4%) were employed at the time of recruitment. Of these, 161 (27.9%) patients started biological therapy, while 416 did not, and these two cohorts form the study population for the current analysis (table 1). Persons starting biological therapy were younger (42.4 years vs 47.0 years), more likely to be smokers (21.3% vs 11.0%) with greater disease activity (Bath Ankylosing Spondylitis Disease Activity Index 5.8 vs 3.3 and Ankylosing Spondylitis Disease Activity Scale 3.4 vs 2.3), poorer function (Bath Ankylosing Spondylitis Functional Index 5.4 vs 2.7) and Bath Ankylosing Spondylitis Global Disease Status 6.7 vs 3.2) (table 1). Persons starting biological therapy also reported higher work impairments at the time of recruitment. They were more likely, during the previous week, to be absent from work (10.9% vs 2.8%), experience greater at-work productivity loss (41.0% vs 20.6%), overall productivity loss (42.3% vs 21.4%) and activity impairment (51.0% vs 24.0%) (table 1). Among those who had not reached normal retirement age at the time of follow-up, there was no significant difference in the proportion who were no longer in employment at follow-up (biological group 5.2% vs non-biological group 4.3%).

**Table 1:** BSRBR-AS study: baseline characteristics of biological and non-biological cohorts

	Biological cohort (n=161)	Non-biological cohort (n=416)	Difference*	95% CI
Age, mean years	42.4	47.0	−4.6	−6.6 to 2.5
Male, %	64.6	67.3	−2.7	−11.3 to 5.9
Disease duration, mean years	7.7	12.3	−4.6	−6.5 to 2.7
Current smokers, %	21.3	11.0	10.2	3.9 to 16.5
BASDAI	5.8	3.3	2.6	2.2 to 2.9
BASFI	5.4	2.7	2.7	2.3 to 3.2
BAS-G§	6.7	3.2	3.4	3.0 to 3.9
CRP (mg/dL)	2.8	2.4	0.4	−0.8 to 1.6
ASDAS	3.4	2.3	1.1	0.9 to 1.3
Physical job, %	44.8	51.0	−6.2	−3.1 to 15.5
WPAI measures (in the last 7 days)				
Absenteeism, %	10.9	2.8	8.1	4.7 to 11.4
Presenteeism, %	41.0	20.6	20.4	15.9 to 24.9
Overall work impairment, %	42.3	21.4	20.9	16.2 to 25.7
Overall activity impairment, %	51.0	24.0	27.0	22.5 to 31.4

*Difference=biological – non-biologic cohort.

ASDAS, Ankylosing Spondylitis Disease Activity Scale; BAS-FI, Bath Ankylosing Spondylitis Functional Index; BAS-G, Bath Ankylosing Spondylitis Global Disease Status; BASDAI, Bath Ankylosing Spondylitis Disease Activity Index; BSRBR-AS, British Society of Rheumatology Biologics register in Axial Spondyloarthritis; CRP, C reactive protein; WPAI, Work Productivity and Activity Impairment.

**Figure 1:**
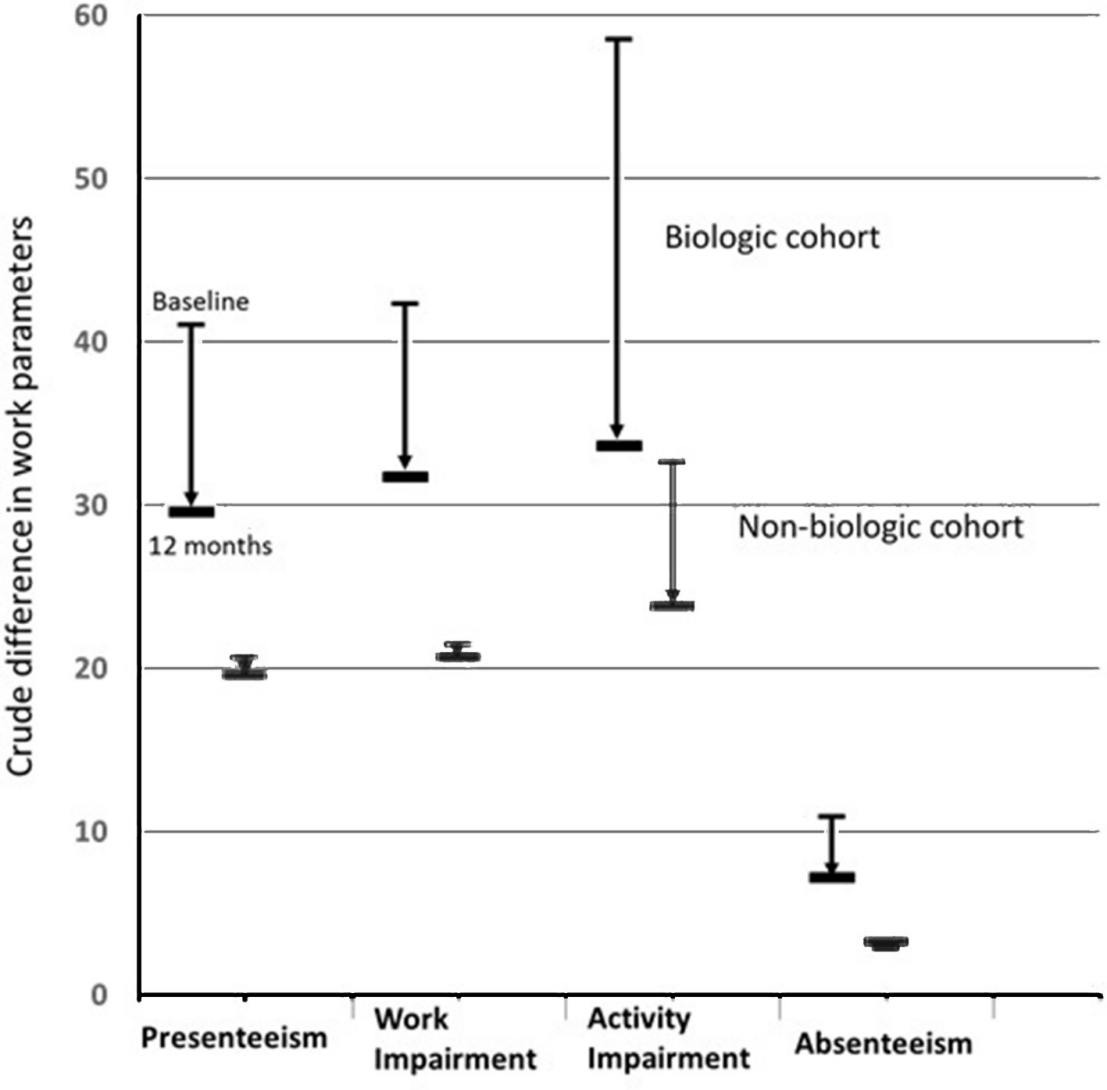
Crude changes in work outcomes after 1 year: BSRBR-AS study. BSRBR-AS, British Society of Rheumatology Biologics register in Axial Spondyloarthritis.

Persons receiving treatment with biological therapy experienced greater improvement in all work outcomes except absenteeism, at 12-month follow-up, compared with persons not receiving biological therapy (figure 1). These improvements remained after propensity score adjustments for differences between the cohorts. Patients on biological therapy demonstrated a significantly greater improvement in presenteeism (−9.4%, 95% CI −15.3% to –3.5%), overall work impairment (−13.9%, 95% CI −21.1% to –6.7%) and overall activity impairment (−19.2%, 95% CI −26.3% to –12.2%). These figures translate into a benefit of over half a day in terms of full productivity per week, 12 months after starting biological therapy. For absenteeism, there was only a small improvement noted in the biological cohort and no significant difference compared with the non-biological cohort (−1.5%, 95% CI −8.0% to 4.9%) (table 2). Nevertheless, despite the greater improvement in these work indices the impact on work was still greater in the biological-treated compared with the non-biological-treated cohort at follow-up (presenteeism biological cohort – non-biological cohort adjusted for factors used in propensity matching: 11.5%, 95% CI 5.5% to 17.5%; overall work impairment: 9.7%, 95% CI 3.6% to 15.9%; overall activity impairment: 9.2%, 95% CI 2.5% to 16.1%).

**Table 2:** BSRBR-AS study: changes in work outcomes between biological and non-biological cohort

WPAI measures (in the last 7 days)	Biological cohort	Non-biological cohort	Mean difference in change*	95% CI
Absenteeism, %	−1.0	0.5	−1.5	−8.0 to 4.9
Presenteeism, %	−11.9	−2.5	−9.4	−15.3 to − 3.5
Overall work impairment, %	−11.9	2.0	−13.9	−21.1 to − 6.7
Overall activity impairment, %	−17.6	1.6	−19.2	−26.3 to − 12.2

+, deterioration; **−**, improvement.

*Difference=biological – non-biological cohort (adjusted for differences using propensity score matching).

WPAI, Work Productivity and Activity Impairment.

The sensitivity analysis conducted showed that the results were insensitive to an unmeasured factor, which would increase the log odds of biological treatment by approximately 1.9 (presenteeism) and 2.0 (overall work impairment and activity impairment).

### Meta-analysis

**Figure 2:**
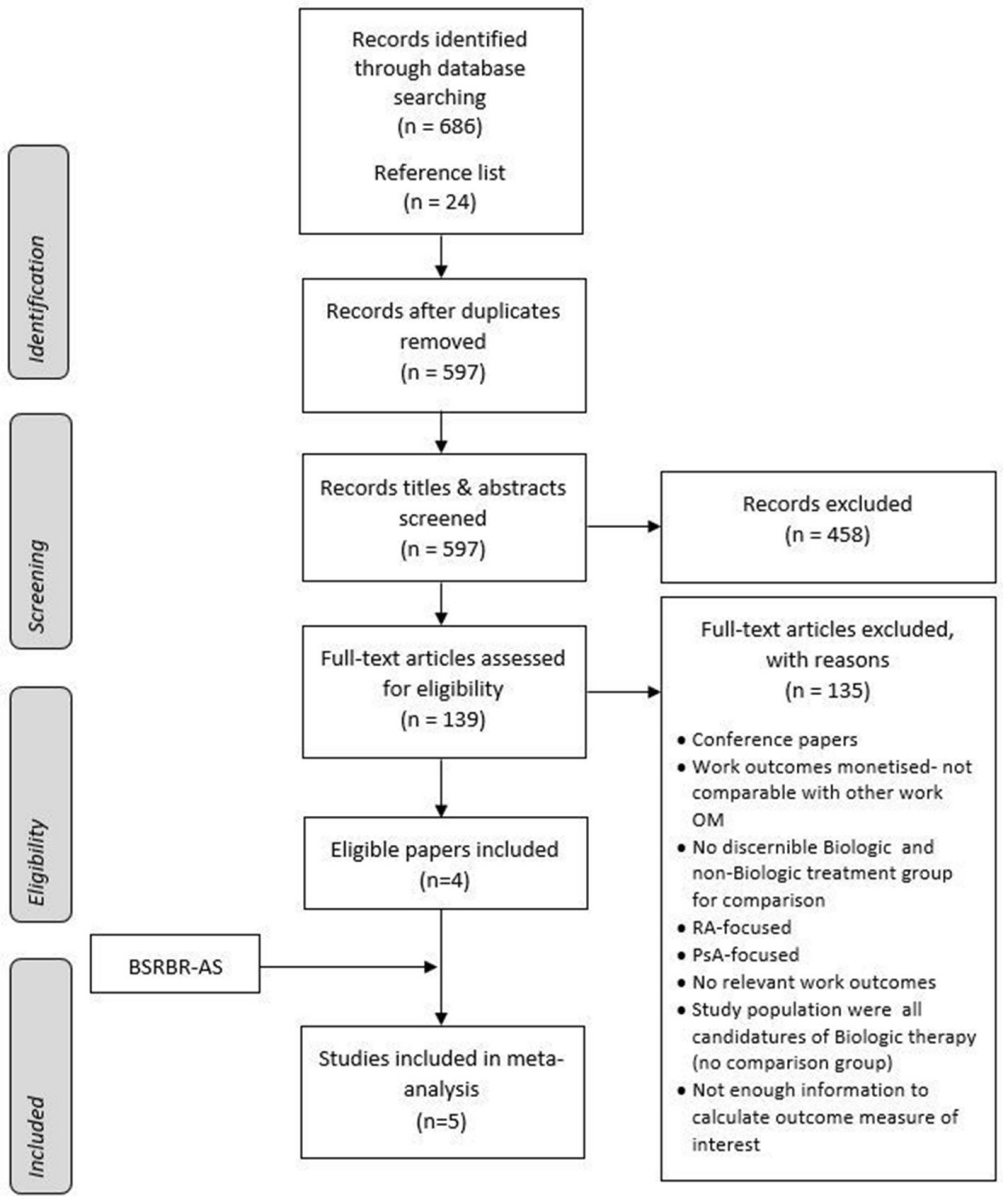
PRISMA flow chart of study selection and inclusion (modified from Moher *et al* 23). BSRBR-AS, British Society for Rheumatology Biologics Register in Axial Spondyloarthritis; OM, outcome measure; PRISMA, Preferred Reporting Items for Systematic Reviews and Meta-Analyses; PsA, psoriatic arthritis; RA, rheumatoid arthritis.

There were 686 publications identified, of which 547 were excluded because they were duplicates, conference abstracts, case reports or not relevant based on title and abstract screening. The remaining 139 publications were retrieved for full-text review. Of the six publications that were identified as potentially eligible, four were included in the meta-analysis; the remaining two were reporting studies already included^25 26^ (figure 2). Results of five studies (four identified in review and the current study) with a total of 1109 participants were therefore included in the meta-analysis. A summary of the characteristics of the studies included is shown in table 3. All of the studies (apart from BSRBR-AS) were RCTs. Three were multinational studies conducted in Europe, Asia and America,^27–29^ and the other was a study conducted within the UK^30^ (table 3). Follow-up ranged from 3 months to 12 months, and the biological drugs used in the trials were infliximab, etanercept and secukinumab. Statistical heterogeneity tests showed minimal to moderate heterogeneity across studies and outcomes (I^2^=0%–66%), and therefore, random effect models were used.

**Table 3:** Characteristics and results of studies eligible for meta-analysis reporting the impact of biological therapy on work participation in patients with axSpA

Authors	Year	Study location	Sampling frame*	Study design	Analysis sample	Sample size†	Biological therapy (N)	Control (N)	Work measure	Reference period of work outcomes	Follow-up
van der Heijde *et al* 27 (ASSERT)	2006	USA, Canada, and Europe	33 centres; adult patients with active AS according to mNYc. Age: mean 39.8 (SD 10.2).	Phase 3, double blind, placebo controlled RCT	Employed patients only	122	94	28	Productivity VAS¶	6 weeks	12 months
Dougados *et al* 28 (EMBARK)	2015	Latin America, Central Europe and Asia	Multicentre; adult patients (≥18 years), satisfied ASAS criteria with non-radiographic sacroiliitis defined as those who did not meet 1984 mNYc. Age: 32.0 (7.8).	Phase 3, double blind, two-period RCT	Employed patients only	123	60	63	WPAI: SHP AS-WIS	1 week	3 months
Deodhar *et al* 29 (MEASURE-1)	2016	Americas, Europe and Asia	65 centres; adult patients (≥18 years) who meet the mNYc for AS and BASDAI ≥4. Age-biological: 40.1 (11.6). Age-placebo: 43.1 (12.4).	Phase 3, double-blind, placebo-controlled RCT.	Employed patients for WPAI (i)–(iii); full population for WPAI (iv).	247	125	122	WPAI: GH	1 week	4 months
Barkham *et al* 30	2010	UK	Adult patients with AS according to mNYc, BASDAI 2/3, VAS ≥40, early morning stiffness ≥45. Age-biologic: 40.8 (9.7). Age-placebo: 39.4 (10.1).	Double-blind, placebo-controlled RCT	Employed patients only	40	20	20	AS-WIS	–	3 months
BSRBR-AS study	2017	UK	Multicentre; adult patients who are biological naïve; meet ASAS criteria for radiographic and non-radiographic axSpA. Age-biological: 47.2 (13.9). Age-non-biological: 53.9 (13.8).	Prospective, register-based data	Employed patients only	577	161	416	WPAI: SHP	1 week	12 months

*Age: mean years (SD).

†Sample size of analysis.

‡WPAI: (i) absenteeism; (ii) presenteeism; (iii) overall work impairment; and (iv) overall activity impairment.

Work Productivity and Activity Impairment: General Health.

AS, ankylosing spondylitis; ASAS, Assessment of SpondyloArthritis International Society; ASSERT, AS Study for the Evaluation of Recombinant Infliximab Therapy; AS-WIS, AS-Work Instability Scale; axSpA, axial spondyloarthritis; BASDAI, Bath Ankylosing Spondylitis Disease Activity Index; BSRBR-AS, British Society of Rheumatology Biologics register in Axial Spondyloarthritis; EMBARK, Study Comparing Etanercept (ETN) Against a Placebo for Etanercept on a Background Nonsteroidal Anti Inflammatory Drug (NSAIDs) in the Treatment of Early Spondyloarthritis (SpA) Patients Who do Not Have X-ray Structural Changes (based on study title registered in NCT); MEASURE 1, Effect of Secukinumab in Patients With Active Anklylosing Spondylitis; mNYc, Modified New York criteria; RCT, randomised controlled trial; VAS, visual analogue scale; WPAI: GH, Work Productivity and Activity Impairment: General Health; WPAI: SHP, Work Productivity and Activity Index: Specific Health Problem.

**Figure 3:**
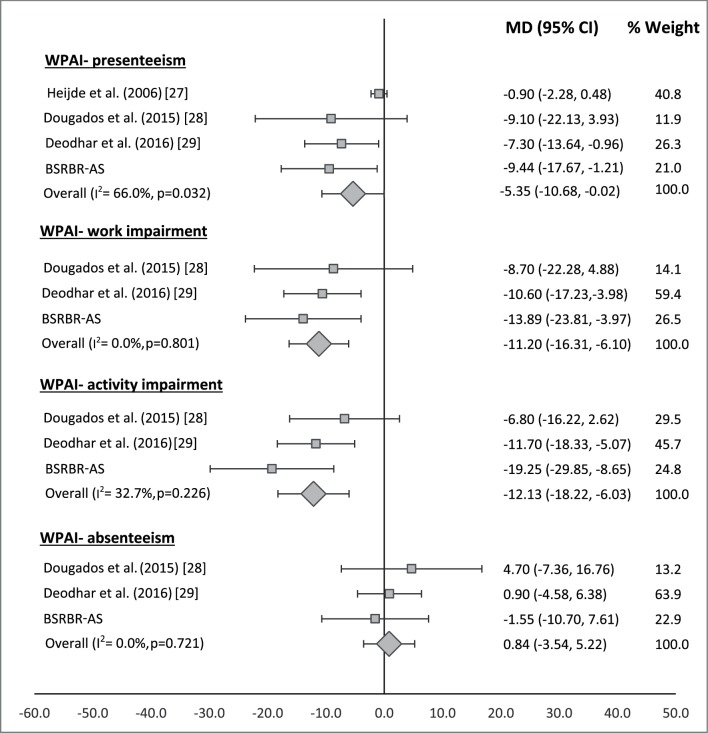
Forest plot comparing changes in WPAI outcomes between patients in the biological and non-biological treatment groups. BSRBR-AS, British Society of Rheumatology Biologics register in Axial Spondyloarthritis; WPAI, Work Productivity and Activity Impairment.

Three studies, with a total of 947 participants (346 biological therapy; 601 non-biological therapy), reported work measures using WPAI. There was a statistically significant difference (in favour of biological therapy groups) for improvement in presenteeism (mean difference (MD)=−5.35, 95% CI −10.68 to –0.02) and overall work impairment (MD=−11.20, 95% CI −16.31 to–6.10), that is, overall at-work productivity improved by 11% more among patients who received biological therapy compared with those on other therapies (figure 3). Furthermore, improvement in self-reported ability to perform daily activities was on average, 12% higher in patients treated with biological therapy (MD=−12.13, 95% CI −18.22 to –6.03) (figure 3). In contrast, there was little improvement in absenteeism in either treatment group; absolute change in biological therapy and non-biologic therapy groups across studies were −2.2% and −6.3%, respectively, and the pooled change in absenteeism was similar in both groups (MD=0.84, 95% CI −3.54 to 5.22) (figure 3).

Only two studies measured work instability that included 163 participants (80 biological; 83 non-biological therapy). On a standardised scale, treatment with biologic therapy was associated with a small improvement on the AS-WIS compared with the non-biological therapy group. However, this difference was not statistically significant (MD=−1.16, 95% CI −2.56 to 0.26) (online supplementary appendix 2).

## Discussion

Patients with axSpA recruited to a national disease register, who started biological therapy showed, on average, significantly greater improvements in work outcomes compared with those not commencing such therapy. Furthermore, pooled data across five cohorts with a total of 1109 patients with axSpA quantified that treatment with biological therapy leads to greater improvements in both work productivity (presenteeism (5%), overall work impairment (11%)) and activity impairment (12%)). There was however no benefit across all studies in relation to absenteeism.

The main strength of the BSRBR-AS study is the large sample size, national coverage and prospective study design. It provides a sample of patients with axSpA recruited from 83 outpatient clinics, including both specialist and non-specialist centres across Great Britain. Participants starting biological therapy have been shown to be similar in characteristics to patients with axSpA recruited to the trials of biological therapies.^31^ Although the effects on work outcomes could be estimated using an RCT, work outcomes are not routinely collected in such trials and they are rarely long enough for change to occur. As this was an observational study, propensity score matching techniques were used to estimate the effect of treatment; this statistical technique addresses ‘confounding by indication’ due to treatment group differences. However, propensity score analysis can only take into account measured factors 32. It is of note that measures of disease activity were not part of the propensity score. However, being highly discriminatory in terms of treatment assignment, this is to be expected, due to the lack of overlap between groups.

Sensitivity analyses suggested only a strong unmeasured confounder could explain the differences observed. Furthermore, it is of note that the results from the observational study (BSRBR-AS) were very similar to the results from the RCTs included in the meta-analysis (with little heterogeneity noted) suggesting that residual confounding has not been a major issue.

To our knowledge, this is the first meta-analysis to quantify the impact of biological therapy on work participation. One earlier systematic review included nine trials and examined the effect of biological treatment on three work outcomes: work status, absenteeism and presenteeism.^15^ It showed that presenteeism decreased by 17%–29% and absenteeism decreased by 8.7–22.3 days over a period of 12 months after commencement of biological therapy. However, these results were based on single group, pre-therapy and post-therapy analysis.^15^

Nevertheless, our review found few studies that investigated the impact of biological therapy on work-related outcomes and relatively small sample sizes in the included trials. Although a substantial improvement in presenteeism and overall work impairment was achieved in the biological cohort, our study shows a persisting gap relative to the non-biological cohort patients with axSpA. This suggests that pharmacological intervention alone is not enough to improve work participation. Overall, the BSRBR-AS study did not demonstrate improvements in absenteeism, and the meta-analysis did not demonstrate any significant differences in the improvements on absenteeism between the biological and non-biological treatment groups. The group who experience presenteeism represent a large proportion of patients with axSpA, and while they are at high risk of absenteeism, this outcome is considerably less common.^33 34^ While we have shown that biological therapy improves presenteeism (relative to not receiving biological therapy), it does not necessarily mean therefore that this leads to an improvement in absenteeism. Our data are consistent with the hypothesis that absenteeism is a late stage in terms of work impairment that is not reversed by biological therapy alone but likely also to be influenced by contextual factors. Zhang *et al* 35 assessed the construct validity of the WPAI questionnaire among patients with rheumatoid arthritis and found absenteeism to correlate the least with health-related outcomes compared with other WPAI domains.^35^ Saidane *et al* 36 also demonstrated that disease severity and disease activity were not associated with absenteeism among people with AS in a cohort study.^36^ We have shown in a previous analysis from BSRBR-AS that presenteeism predicts future absenteeism, which predicts future work loss. Taken together, these results emphasise that biological therapy may be less effective at improving work outcome when given late in the natural history of the condition.^37^ Cost-effectiveness analysis often consider costs from a payer’s perspective (in the UK, the NHS) rather than considering the wider societal costs (and benefits) associated with treatment. Although presenteeism is an important outcome, it would be interesting to extend existing cost-effectiveness estimates to take important work outcomes into account. However, these data were not available and is therefore beyond the scope of the current study.

We conclude that there is consistent evidence, across different study designs, that treatment with biological therapy significantly and meaningfully improves work productivity and activity impairment in people with axSpA. However, even with the improvements observed with biological therapy, there is still a substantial impact on work. Future studies in axSpA should include assessment of work outcomes as standard, ensuring a greater evidence base around pharmacological and non-pharmacological approaches to improving work outcomes in patients with axSpA.
