# Supplementary material for: Impact of biological therapy on work outcomes in patients with axial spondyloarthritis: results from the British Society for Rheumatology Biologics Register (BSRBR-AS) and meta-analysis
Source: Ann Rheum Dis. 2018 Aug 3;77(11):1578–84. doi: 10.1136/annrheumdis-2018-213590 (PMC6225801; doi:10.1136/annrheumdis-2018-213590)
Supplement: Supplementary file 1 [file annrheumdis-2018-213590supp001.docx]

# Appendix 1

#1: (axial spondyloarthritis).mp OR (ankylosing spondylitis).mp OR axspa.mp OR as.mp OR spondyloarthritis/exp OR spondyloarthritis.mp.

#2: (biologic$ ADJ5 treatment).mp OR (biologic$ ADJ5 therapy).mp OR (biologic$ ADJ5 agent).mp OR (anti$tnf).mp OR (tnf inhibitor).mp OR (anti$tumo$r necrosis factor).mp OR etanercept/exp OR etanercept.mp OR infliximab/exp OR infliximab.mp OR anakinra/exp OR anakinra.mp OR adalimumab/exp OR adalimumab.mp OR abatacept/exp OR abatacept.mp OR golimumab/exp OR golimumab.mp OR rituximab/exp OR rituximab.mp OR certolizumab/exp OR certolizumab.mp OR tocilizumab/exp OR tocilizumab.mp OR secukinumab/exp OR secukinumab.mp

#3: (work disability).mp OR productivity/exp OR productivity.mp OR employability/exp OR employability.mp OR (working ability).mp OR absenteeism/exp OR absenteeism.mp OR (sick leave).mp OR presenteeism/exp OR presenteeism.mp OR (sickness absence).mp OR (work instability).mp OR (return to work).mp OR RTW/exp OR RTW OR job/exp OR job.mp OR work/exp OR work.mp OR employment/exp OR employment.mp OR work impairment.mp OR occupation

#1 AND #2 AND #3

**Figure S1**. Search strategy (Ovid MEDLINE, EMBASE, Evidence Based Medicine (EBM), and Cochrane Library)

# Appendix 2

**Authors**

**% Weight**

**MD (95% CI)**

**AS-WIS**

**Figure S2.** Forest plot showing change in AS-Work Instability Scale (AS-WIS) between patients in the TNFi and non‑TNFi treatment group
